# Antibiotics resistance in El Tor *Vibrio cholerae* 01 isolated during cholera outbreaks in Mozambique from 2012 to 2015

**DOI:** 10.1371/journal.pone.0181496

**Published:** 2017-08-08

**Authors:** Liliana Candida Dengo-Baloi, Cynthia Amino Semá-Baltazar, Lena Vania Manhique, Jucunu Elias Chitio, Dorteia Luísa Inguane, José Paulo Langa

**Affiliations:** Instituto Nacional de Saúde, Maputo, Moçambique; Beijing Institute of Microbiology and Epidemiology, CHINA

## Abstract

**Rationale:**

Mozambique has recorded cyclically epidemic outbreaks of cholera. Antibiotic therapy is recommended in specific situations for management and control of cholera outbreaks. However, an increase in resistance rates to antibiotics by *Vibrio cholerae* has been reported in several epidemic outbreaks worldwide. On the other hand, there are few recent records of continuous surveillance of antibiotics susceptibility pattern of *V*. *cholerae* in Mozambique.

**Goals:**

The purpose of this study was to evaluate antibiotics resistance pattern of *Vibrio cholerae* O1 Ogawa isolated during Cholera outbreaks in Mozambique to commonly used antibiotics.

**Methodology:**

We analyzed data from samples received in the context of surveillance and response to Cholera outbreaks in the National Reference Laboratory of Microbiology from the National Institute of Health of Mozambique, 159 samples suspected of cholera from cholera treatment centers of, Metangula (09), Memba (01), Tete City (08), Moatize (01), Morrumbala (01) districts, City of Quelimane (01), Lichinga (06) and Nampula (86) districts, from 2012 to 2015. Laboratory culture and standard biochemical tests were employed to isolate and identify *Vibrio cholerae*; serotypes were determined by antisera agglutination reaction in blade. Biotype and presence of important virulence factors analysis was done by PCR. Antibiotics susceptibility pattern was detected by disk diffusion method Kirby Bauer. Antibiotic susceptibility and results were interpreted by following as per recommendations of CLSI (Clinical and Laboratory Standards Institute) 2014. All samples were collected and tested in the context of Africhol Project, approved by the National Bioethics Committee for Health.

**Results:**

Among isolates from of *Vibrio cholerae* O1 El Tor Ogawa resistance to Sulphamethoxazole-trimethropim was 100% (53/53) to Trimethoprim-, being 100% (54/54) for Ampicillin, 99% (72/74) for Nalidixic Acid, 97% (64/66) to Chloramphenicol, 95% (42/44) for Nitrofurantoin and (19/20) Cotrimoxazole, 83% (80/97) Tetracycline, 56% (5/13) Doxycycline, 56% (39/70) Azithromycin and 0% (0/101) for Ciprofloxacin. PCR analysis suggested strains of *V*. *cholerae* O1 being descendants of the current seventh pandemic *V*. *cholerae* O1 CIRS 101 hybrid variant. The *V*. *cholerae* O1 currently causing cholera epidemics in north and central Mozambique confirmed a CTXΦ genotype and a molecular arrangement similar to the *V*. *cholerae* O1 CIRS 101.

**Conclusion:**

Although *V*. *cholerae* infections in Mozambique are generally not treated with antibiotics circulating strains of the bacteria showed high frequency of in vitro resistance to available antibiotics. Continuous monitoring of antibiotic resistance pattern of epidemic strains is therefore crucial since the appearance of antibiotic resistance can influence cholera control strategies.

## Introduction

*Vibrio cholerae* toxin is the virulence factor causing cholera disease, which is characterized by a secretory acute diarrhea. Cholera can lead to severe dehydration and death within hours if not promptly treated. Cholera constitutes a serious public health problem in many parts the world [1,2].

The seventh pandemic of cholera reached Africa in the 70’s, arrived in Mozambique in 1973 with cases reported until 1985. Cholera resurfaced in 1989 with over 3,600 cases reported, caused by *V*. *cholerae* O1 El Tor serotype Inaba (with some cases of Ogawa), susceptible to Tetracycline, Chloramphenicol and sulfadiazine. [3].

Mozambique continued on experiencing recurrent outbreaks of cholera, in different parts of the country, with different spatial pattern from year to year. Increase in antibiotic resistance including resistance to recommended antibiotic for treatment has also been reported [4,5]. The last decades have seen a growing trend in antimicrobial resistance in Mozambique, previous researches have reported *V*. *cholera*e Ogawa O1 El Tor drug resistance in the last decade for Maputo [4–6], Zambezia and Tete [5].

Antimicrobial resistance of *V*. *cholerae* O1 El Tor is of interest because it’s becoming a serious public health problem for many African countries [7]. Therapy with effective antimicrobial agents significantly reduces the duration of diarrhea and hospitalization, reduces the volume of watery feces and need for maintenance fluids. The duration of fecal excretion of *V*. *cholerae* is also decreased, reducing transmission of infection to family members, as well as nosocomial infections [8].

Increasing drug resistance is well known and usually varies from one place to another. *V*. *cholerae* becomes drug resistant by exporting drugs through efflux pumps, chromosomal mutations or developing genetic resistance via the exchange of conjugative plasmids, conjugative transposons, integrons or self- transmissible chromosomally integrating *sxt* elements. In addition, *V*. *cholerae*, as an environmental organism, have means to acquire resistance genes from intimate contact with intrinsically resistant environmental bacteria, through mobile genetic elements. *V*. *cholerae* is able to share these antibiotic resistance genes with other bacteria; and once in the human gut, the bacteria may share these resistance traits with commensals or other enteric pathogens, what complicates antibiotic therapy of many infections [2].

Since antibiotic therapy is recommended in specific situations for management and control of cholera outbreaks, monitoring *V*.*cholerae* resistance is important for public health. The aim of this study was to describe spatiotemporally the antimicrobial resistance pattern of *Vibrio cholerae* O1 El Tor Ogawa isolated in patients admitted to cholera treatment centers (CTCs) and diarrheic disease treatment centers (CTDDs) during outbreak investigations in Mozambique, from 2012 to 2015.

## Material and methods

### Sample collection and transportation

Rectal swabs from suspected cholera cases were collected during Cholera outbreaks from CTCs and CTDD’s from 2012 to 2015 by local laboratory technicians and transported to local laboratories in Cary Blair medium, prepared from dehydrated media. These samples were collected after patient’s stabilization and before antimicrobial administration.

### Culture and identification

Laboratory technicians, at provincial level, when possible, *Vibrio cholerae* were identified by standard culture methods; and sent to National Reference Laboratory of Microbiology (NRLM), at central level, for serological confirmation, Antimicrobial susceptibility test (AST) and a double multiplex PCR for mobilome profile analysis.

In the laboratory, samples were enriched in APA broth, then cultured in TCBS media, and suspected colonies were submitted to standard biochemical tests; when positive, colonies were submitted to serological tests for *Vibrio cholerae* TM Difco BD Poly and Vibrio *cholerae* TM Difco BD Ogawa (Denka Seiken, Tokyo, Japan).

### Antimicrobial susceptibility testing (Ast)

Serologically positive samples were submitted to AST by Kirby Bauer diffusion method [9] for commonly used and recommended antibiotics, namely, Nalidixic acid, Ampicillin, Sulphamethoxazole-trimethropim (SXT), Tetracycline, Chloramphenicol, Nitrofunrantoin, Azithromycin, Cotrimoxazole and Doxycycline, according to laboratory’s antibiotic discs availability at the moment. Results from AST were interpreted using CLSI (2014).

### PCR analyses

Representatively, positive isolates on serological tests were submitted to a double multiplex PCR essay, using five completely sequenced reference strains as positive controls (MJ1236.N1696, B33, CIRS101 and MO10), for the presence of R*stR*, *ctxB*, and *tcpA genes*, genetic markers for El Tor Biotype [10].

### Ethical considerations

Analyzed data was from samples collected during Africhol Project, the African Cholera Surveillance Network multi-centric project consisting of an 11 African countries consortium and non-governmental organizations, aiming to collect epidemiological and microbiological information for the occurrence of cholera in Africa to advise for control and preventive measures (http://africhol.org/), which surveillance protocol was approved by the Mozambican National Bioethics Committee for Health. During the study, all participants gave an informed written consent.

We analyzed Africhol data from the laboratory records, anonymously (all samples were codified upon entrance), there was no direct intervention or interaction with human subjects and neither identifiable private information.

## Results

**Fig 1:**
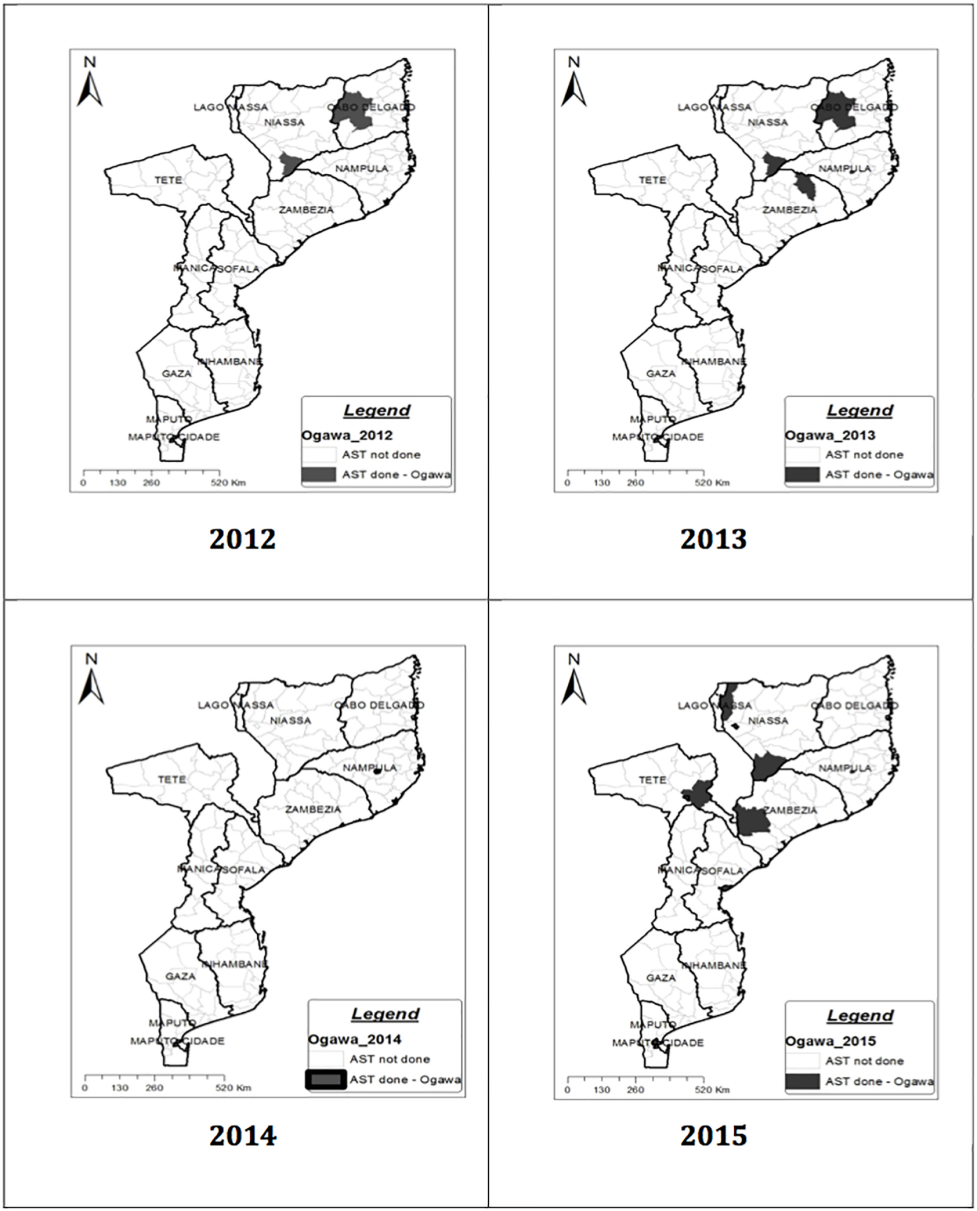
AST per district and per year, for *V*. *cholerae* O1 El Tor Ogawa isolated during cholera outbreaks in Mozambique from 2012 to 2015. In 2012 (Cuamba district in Niassa province and Montepuez *district* in Cabo Delgado province), 2013 (Cuamba district in Niassa province, Pemba city and Montepuez *districts* in Cabo Delgado province, Nampula city *district* in Nampula province and Alto-Molócue *district* in Zambezia province), 2014 (Nampula city *district* in Nampula province), 2015 (Lichinga city, Lago and Cuamba *districts* in Niassa province, Nampula city *district* in Nampula province and Morrumbala and Quelimane city *districts* in Zambézia province, Tete city and Moatize *districts* in Tete province, Beira city *district* in Sofala province and Matola city *district* in Maputo province). AMP- Ampicillin; TE-Tetracycline; NA- Nalidixic Acid; C-Chloramphenicol; CIP-Ciprofloxacin; SXT- Sulphamethoxazol-trimethropim; F- Nitrofurantoin; AZM- Azithromycin; rstR, ctxB and tcpA- *Vibrio cholerae* virulence genes; TLC-RS1, CORE-RTX and TCL-RS2—primers for the presence of CTX ϕ on chromosome 1; Chr II—Chromosome 2

Mozambique reported 27 outbreaks in 22 districts (of 145), on 07 of 10 provinces (Fig 1), from 2012 to 2015; and 1522 samples of suspected Cholera cases were received at the National Reference Laboratory of Microbiology, 510 confirmed as *V*. *cholerae* serogroup O1 biotype El Tor serotype Ogawa. From these 510, 159 were submitted to AST.

Table 1 shows *V*. *cholerae* O1 El Tor Ogawa antibiotic resistance during epidemics, between 2012 and 2015. In 04 isolates tested on 2012’s outbreaks we found no resistance to Ciprofloxacin and Azithromycin, low levels of resistance to Tetracycline (25%), and 100% resistance to Ampicillin, Nalidixic Acid, Chloramphenicol, SXT and Doxycyclin.

**Table 1:** Antibiotic resistance in *V*. *cholerae* O1 El Tor Ogawa isolated during cholera outbreaks in Mozambique from 2012 to 2015.

Antimicrobial agent			2012	2013	2014	2015	% (R)
Ampicillin	AMP	10μg	100%	100%	100%	100%	100%
Tetracycline	TE	30μg	25%	32%	44%	100%	50%
Nalidixic Acid	NA	30μg	100%	100%	100%	100%	100%
Chloramphenicol	C	10ug	100%	58%	97%	100%	89%
Ciprofloxacin	CIP	5μg	0%	0%	0%	0%	0%
Trimethropim-Sulphamethoxazol	SXT	23.75/1.25μg	100%	100%	0%	100%	75%
Nitrofurantoin	F	300μg	n.t.	n.t.	94%	100%	97%
Azithromycin	AZM	15μg	0%	0%	n.t.	39%	13%
Cotrimoxazol	CTX	30μg	n.t.	n.t.	n.t.	95%	95%
Doxycyclin	DO	30μg	100%	11%	n.t.	n.t.	56%
number of isol ates tested per year	-	-	n = 4	n = 19	n = 35	n = 100	-

n.t. = not tested

On 2013, we tested 19 isolates with no resistance to Ciprofloxacin, low resistance to Doxycyclin (11%), increased resistance to Tetracycline (32%), resistance to Chloramphenicol (58%) and 100% resistance to Ampicillin, Nalidixic Acid, SXT and Azithromycin.

In 2014, for 35 isolates, we found no resistance to Ciprofloxacin and SXT, increased resistance to tetracycline (44%), high resistance to Nitrofurantoin (94%), Chloramphenicol (97%) and 100% to Ampicillin and Nalidixic acid.

For 2015 isolates (n = 100) we found 100% resistance for Ampicillin, Tetracycline, Nalidixic acid, Chloramphenicol, SXT and Nitrofurantoin; 95% resistance to Cotrimoxazole and no resistance to Ciprofloxacin.

**Fig 2:**
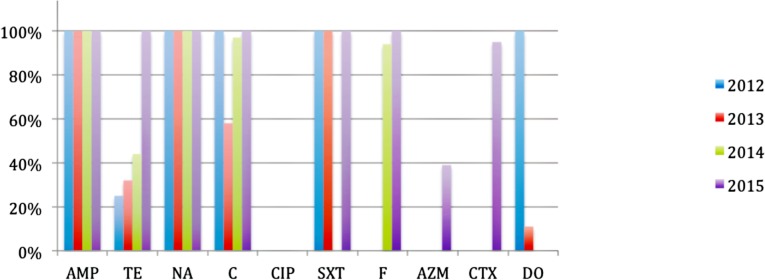
Percentage of antibiotic resistance in *V*. *cholerae* O1 El Tor Ogawa isolated during cholera outbreaks in Mozambique from 2012 to 2015. AMP- Ampicillin; TE-Tetracycline; NA- Nalidixic Acid; C-Chloramphenicol; CIP-Ciprofloxacin; SXT- Sulphamethoxazol-trimethropim; F- Nitrofurantoin; AZM- Azithromycin.

Tendency of resistance from 2012 to 2015 (Fig 2) indicates an increase in antimicrobial resistance for Tetracycline, Nitrofurantoin, and Azithromycin. A sudden decrease and subsequent increase of resistance in Chloramphenicol and SXT, decrease in Doxycycline from 2012 to 2013, 95% resistance in 2015 for Cotrimoxazole and 100% resistance along the years for Ampicillin and Nalidixic Acid. Laboratory analyses also shows that *V*. *cholerae* O1 El Tor Ogawa isolated during cholera outbreaks in Mozambique, have no resistance to Ciprofloxacin.

Overall *V*. *cholerae* from 2012 to 2015 outbreaks had 100% resistance to Ampicillin and Nalidixic Acid, 97% to Nitrofurantoin, 95% to Cotrimoxazole, 89% to Chloramphenicol, 75% to SXT, 56% to Doxycycline, 50% to Tetracycline, 13% to Azithromycin and 0% to Ciprofloxacin.

Genetic screening by PCR in 58 isolates revealed three important El Tor epidemic markers, *ctxA*, *rstR2*, and t*cpA*, and the presence of CTX ϕ on chromosome 1 instead of chromosome 2, confirming the profile found in *V*. *cholerae* O1 El Tor variants B33 and CIRS 101. (Table 2)

**Table 2:** CTX ϕ cluster analysis of *Vibrio cholerae* O1 El Tor Ogawa isolated during cholera outbreaks in Mozambique from 2012 to 2015. Showing a classic signature of *Vibrio cholerae* O1 El Tor variants B33 and CIRS 101. AMP- Ampicillin; TE-Tetracycline; NA- Nalidixic Acid; C-Chloramphenicol; CIP-Ciprofloxacin; SXT- Sulphamethoxazol-trimethropim; F- Nitrofurantoin; AZM- Azithromycin; rstR, ctxB and tcpA- *Vibrio cholerae* virulence genes; TLC-RS1, CORE-RTX and TCL-RS2—primers for the presence of CTX ϕ on chromosome 1; Chr II—Chromosome 2.

			AMP	TE	NA	C	CIP	SXT	AZM	DO	Mobilome							
Ano	testados	proveniencia	10μg	30μg	30μg	10ug	5μg	23.75/1.25μg	15μg	30μg	ICE	VSP-II	TLC	Kappa	GI-12	GI-14	GI-15	Profile
1975	N16961	India	-	-	-	-	-	-	-	-	-	+	+	-	-	-	-	A
1992	MO10	India	-	-	-	-	-	-	-	-	SXT	+	+	+	-	-	-	E
1994	MJ-1236	Bangladesh	-	-	-	-	-	-	-	-	ICE*Vch*Ban9	+	-	+	+	+	-	D
2002	CIRS101	India	-	-	-	-	-	-	-	-	ICE*Vch*Ind5	+ (tr)	+	-	-	-	-	B
2012	5	Nampula	R	I	R	R	S	R	S	I	ICE*Vch*Ind5	+ (tr)	+	+	-	-	-	B + K
2013	3	Cabo Delgado	R	I	R	I	S	R	S	S	ICE*Vch*Ind5	+ (tr)	+	+	-	-	-	B + K
2013	1	Cabo Delgado	R	S	R	I	S	R	S	S	ICE*Vch*Ind5	+ (tr)	+	+	-	-	-	B + K
2013	2	Nampula	R	I	R	R	S	R	S	S	ICE*Vch*Ind5	+ (tr)	+	+	-	-	-	B + K
2013	1	Nampula	R	I	R	I	S	R	S	S	ICE*Vch*Ind5	+ (tr)	+	+	-	-	-	B + K
2013	1	Nampula	R	R	R	R	S	R	S	S	ICE*Vch*Ind5	+ (tr)	+	+	-	-	-	B + K
2013	1	Cabo Delgado	R	I	R	R	S	-	S	S	ICE*Vch*Ind5	+ (tr)	+	+	-	-	-	B + K
2013	1	Cabo Delgado	R	S	R	S	S	R	S	S	ICE*Vch*Ind5	+ (tr)	+	+	-	-	-	B + K
2013	1	Cabo Delgado	R	I	R	I	S	R	S	S	ICE*Vch*Ind5	+ (tr)	+	+	-	-	-	B + K
2013	3	Cabo Delgado	R	R	R	R	S	R	S	S	ICE*Vch*Ind5	+ (tr)	+	+	-	-	-	B + K
2013	5	Cabo Delgado	R	I	R	R	S	R	S	S	ICE*Vch*Ind5	+ (tr)	+	+	-	-	-	B + K
2013	3	Niassa	R	R	R	R	S	R	S	-	ICE*Vch*Ind5	+ (tr)	+	+	-	-	-	B + K
2013	1	Zambézia	R	I	R	I	S	R	S	-	ICE*Vch*Ind5	+ (tr)	+	-	-	-	-	B
2013	2	Zambézia	R	R	R	R	S	R	S	-	ICE*Vch*Ind5	+ (tr)	+	-	-	-	-	B
2014	17	Nampula	R	R	R	R	S	S	-	-	ICE*Vch*Ind5	+ (tr)	+	-	-	-	-	B
2014	11	Sofala	R	R	R	R	S	S	-	-	ICE*Vch*Ind5	+ (tr)	+	-	-	-	-	B
			AMP	TE	NA	C	CIP	SXT	AZM	DO	CTXϕ cluster organizatiom							
Year	tested isolates	Origin	10μg	30μg	30μg	10ug	5μg	23.75/1.25μg	15μg	30μg	CtxB	rstR	tcpA	TLC-RS1	CORE-RTX	TCL-RS2	Chr II	
2012	5	Nampula	R	I	R	R	S	R	S	I	cla	cla	Et	3000	1542	1740	0	
2013	3	Cabo Delgado	R	I	R	I	S	R	S	S	cla	cla	Et	3000	1542	1740	0	
2013	1	Cabo Delgado	R	S	R	I	S	R	S	S	cla	cla	Et	3000	1542	1740	0	
2013	2	Nampula	R	I	R	R	S	R	S	S	cla	cla	Et	3000	1542	1740	0	
2013	1	Nampula	R	I	R	I	S	R	S	S	cla	cla	Et	3000	1542	1740	0	
2013	1	Nampula	R	R	R	R	S	R	S	S	cla	cla	Et	3000	1542	1740	0	
2013	1	Cabo Delgado	R	I	R	R	S	-	S	S	cla	cla	Et	3000	1542	1740	0	
2013	1	Cabo Delgado	R	S	R	S	S	R	S	S	cla	cla	Et	3000	1542	1740	0	
2013	1	Cabo Delgado	R	I	R	I	S	R	S	S	cla	cla	Et	3000	1542	1740	0	
2013	3	Cabo Delgado	R	R	R	R	S	R	S	S	cla	cla	Et	3000	1542	1740	0	
2013	5	Cabo Delgado	R	I	R	R	S	R	S	S	cla	cla	Et	3000	1542	1740	0	
2013	3	Niassa	R	R	R	R	S	R	S	-	cla	cla	Et	3000	1542	1740	0	
2013	1	Zambézia	R	I	R	I	S	R	S	-	cla	cla	Et	3000	1542	1740	0	
2013	2	Zambézia	R	R	R	R	S	R	S	-	cla	cla	Et	3000	1542	1740	0	
2014	17	Nampula	R	R	R	R	S	S	-	-	cla	cla	Et	3000	1542	1740	0	
2014	11	Sofala	R	R	R	R	S	S	-	-	cla	cla	Et	3000	1542	1740	0	

## Discussion

The current study found increasing antibiotic resistance in *Vibrio cholerae* O1 El Tor Ogawa isolated from Cholera outbreaks from 2012 and 2015 in Mozambique to Tetracycline, Trimethropim-sulphamethoxazol, Chloramphenicol and Nitrofurantoin.

Increasing resistance to tetracycline was consistent with data from Zambia [11], urban and rural Bangladesh [12]; DRC [13]; Nigeria [14] and South Mozambique [4,6]. However, decreasing resistance to tetracycline have been reported in other places, such as Calcutta [15], East Delhi [16] and Ghana [17] and susceptibility to tetracycline, in Puduchuary in India [18], Haiti [19] and north India [20].

Our results indicating resistance to Nitrofurantoin and SXT, match to those observed in earlier studies in Zambia [11], and DRC [13]; and SXT resistance also in a Mozambique rural area [4], and in north India [20].

SXT resistance pattern was in accord with Folgosa et al ten years ago in South and Central Mozambique, having along studied years, all isolates resistant to this antibiotic disc except for one, that was sensitive to it [5].

Another important finding was that isolates were 100% sensitive to Ciprofloxacin activity throughout the studied years; In agreement with Folgosa [5] in 3 provinces in Mozambique (Maputo, Zambezia and Tete), Gujral in Maputo city and province; although these results differ from some published studies in Calcutta [15], East Delhi [16], Nigeria [14], Haiti [19], Iran [21], urban and rural Bangladesh [12], Ghana [17] and Indonesia [22] with resistance or increasing resistance to Ciprofloxacin.

Along the years, isolates in our study were 100% resistant Ampicillin, which is consistent with results from Calcutta [15], Zambia [11] and in contrast with Mozambique in the last decade, North India [20] and Indonesia [22].

Resistance to Nalidixic acid for tested isolates in the study was 100%, being consistent with data obtained from Iran [21], Haiti [19], Mozambique South rural area [4], and an increasing resistance in Calcutta [15,17]. Conversely, in East Deli [16] and urban South Mozambique [6] was found susceptibility to Nalidixic acid in tested isolates.

In our study, isolates were 100% resistant to Chloramphenicol in 2012, registered a sudden drop of nearly 50% in 2013 with subsequent increase; this behavior was seen in Calcutta [15] and Zambia [11]; while susceptibility to Chloramphenicol was found in East Delhi [16]; and Mozambique [4,6].

Isolates from 2015 were 95% Resistant to Cotrimoxazole, like in Mozambique in 2007 [6], Calcutta [15], Zambia [11], Iran [21], Ghana [17] and East Delhi [16].

Regarding Doxycycline and Azythromycin, recomended drugs for Cholera control by WHO, like in Zambia [11] and Ghana [17], there was some resistance for Doxycyclin and like in urban and rural Bangladesh [12] and Haiti [23], 2012–2013 isolates were susceptible to Azythromycin, while 2015’s like Ghana [17] presented with some resistance.

PCR screening revealed the same virulence genes found in Mozambique by Folgosa [5] and [24], Cholera toxin *ctxA* and toxin-coregulated pilus, *tcpA*, contained by the majority of *V*. *cholerae* O1 strains, confirming the profile found in *V*. *cholerae* O1 El Tor variants B33 and CIRS 101 (CIRS 101 from Bangladesh). Diferent mobilome profiles have been reported for other African countries where *V cholerae* occurs and unique Mozambique’s profile may be associated with different antibiotic resistance profile.

Due to the plasticity of *V*. *cholerae* resulting in the constant emergence of variants, surveillance and characterization of outbreak strains, and their antibiotic resistance determinants, is essential on defining the complex scenario of cholera in this continent as well as worldwide [14].

These findings may be somewhat limited since AST was done according to laboratory discs availability at the moment, usual outbreak scenario where not all antibiotics are tested for each isolate; that could be different on an established antibiotics resistance surveillance scenario, where you predict your demand and therefore your supply. This disabled not only the evaluation of susceptibility profiles for different affected areas and testing for all affected areas but the evaluation of the resistance pattern throughout studied years for Cotrimoxazole, Nitrofurantoin, Azithromycin and Doxycyclin, last two antibiotics specially important because of their reported antimicrobial activity on *V*. *cholerae* in previous years and currently in other countries where this pathogen occurs.

Although, serological testing and PCR analyses shows for all strains the same serotype and same genetic similarity, a genetic profile coming from the same clonal origin, it is possible, therefore, to assume a similar resistance pattern for each year.

## Conclusion

In general, therefore, it seems that antibiotic resistance profile of *V*. *cholerae* regarding same serotype and same year varies in different countries and this study strengthens the importance of having local antibiotic choice based on an updated AST local report.

With a highly frequent and increasing resistance, the current data highlights the importance to monitor antimicrobial resistance in epidemic strains, since the appearance of antimicrobial resistance to commonly used and recommended antibiotics will influence Cholera national control strategies.
